# The Role of Vitamin D in Inflammatory Bowel Diseases: From Deficiency to Targeted Therapeutics and Precise Nutrition Strategies

**DOI:** 10.3390/nu17132167

**Published:** 2025-06-29

**Authors:** Giuseppe Dell’Anna, Fabrizio Fanizzi, Alessandra Zilli, Federica Furfaro, Virginia Solitano, Tommaso Lorenzo Parigi, Ambra Ciliberto, Jacopo Fanizza, Francesco Vito Mandarino, Lorenzo Fuccio, Antonio Facciorusso, Gianfranco Donatelli, Mariangela Allocca, Sara Massironi, Vito Annese, Laurent Peyrin-Biroulet, Silvio Danese, Ferdinando D’Amico

**Affiliations:** 1 Gastroenterology and Gastrointestinal Endoscopy Division, IRCCS San Raffaele Hospital, Via Olgettina 60, 20132 Milan, Italyfurfaro.federica@hsr.it (F.F.); fanizza.jacopo@hsr.it (J.F.); mandarino.francesco@hsr.it (F.V.M.); damico.ferdinando@hsr.it (F.D.); 2 Gastroenterology and Gastrointestinal Endoscopy Division, IRCCS Policlinico San Donato, Piazza Edmondo Malan 2, 20097 San Donato Milanese, Italy; vito.annese@grupposandonato.it; 3 Unité d’Endoscopie Interventionnelle, Hôpital Privé des Peupliers, 75013 Paris, France; donatelligianfranco@gmail.com; 4 Faculty of Medicine and Surgery, Vita-Salute San Raffaele University, Via Olgettina 56, 20132 Milan, Italy; 5 Gastroenterology Unit, Istituti Ospedalieri Bergamaschi, 24046 Zingonia (Bergamo), Italy; 6 Gastroenterology Unit, IRCCS Azienda Ospedaliero-Universitaria di Bologna, 40138 Bologna, Italy; 7 Gastroenterology Unit, Faculty of Medicine and Surgery, University of Salento, 73100 Lecce, Italy; 8 Department of Clinical Medicine and Surgery, University of Naples Federico II, 80138 Naples, Italy; 9 INSERM NGERE, Department of Gastroenterology, INFINY Institute, CHRU Nancy, 54500 Vandœuvre-lès-Nancy, France; peyrinbiroulet@gmail.com

**Keywords:** vitamin D, inflammatory bowel disease, Crohn’s disease, ulcerative colitis, biologics, diet, personalized medicine, micronutrient deficiency

## Abstract

Background: Vitamin D plays a crucial role in immune modulation, gut barrier integrity, and inflammation regulation, making it highly relevant in inflammatory bowel disease (IBD). IBD patients often exhibit vitamin D deficiency, which has been linked to increased disease activity, impaired mucosal healing, and a higher risk of complications, including infections and osteoporosis. Methods: This review examines the biological functions of vitamin D in maintaining intestinal homeostasis, particularly in the context of IBD. It discusses the prevalence and consequences of vitamin D deficiency in IBD, including its potential to exacerbate disease progression, impair treatment efficacy, and negatively impact long-term health outcomes. Furthermore, therapeutic strategies to address vitamin D deficiency are explored, including supplementation approaches, dosing strategies, and precision nutrition interventions that aim to personalize vitamin D management based on individual patient needs and disease characteristics. Results: By synthesizing the latest evidence, this review highlights the critical role of vitamin D in IBD management, underlining how optimal vitamin D levels may not only improve disease control but also enhance patient quality of life and reduce the risk of long-term complications associated with the disease. Conclusions: Understanding the importance of vitamin D in IBD could help refine treatment strategies and promote better health outcomes for affected individuals.

## 1. Introduction

Inflammatory bowel diseases (IBD), including Crohn’s disease (CD) and ulcerative colitis (UC), are chronic, relapsing inflammatory disorders of the gastrointestinal tract. A complex interplay of genetic predisposition, immune dysregulation, environmental triggers, and alterations in the gut microbiota drives IBD’s development [1]. Over recent decades, the incidence of IBD has risen globally, contributing to a growing public health burden and significantly affecting patients’ quality of life [2].

Among the environmental and modifiable factors under investigation, vitamin D status has emerged as a particularly relevant element in the context of IBD. Beyond its well-established role in calcium homeostasis and bone metabolism, vitamin D is increasingly recognized as a pivotal immunomodulatory agent with far-reaching implications for gut health. It exerts influence on both innate and adaptive immune responses, modulating the activity of dendritic cells, macrophages, and T lymphocytes. Additionally, vitamin D supports the integrity and function of the epithelial barrier, a critical defense against luminal antigens and pathogens, thereby reducing the risk of mucosal injury and permeability. Moreover, it contributes to the regulation of key inflammatory pathways, including the inhibition of pro-inflammatory cytokines, such as TNF-α and IL-6, and the promotion of anti-inflammatory mediators like IL-10. These mechanisms are highly pertinent to the pathophysiology of IBD, where a dysregulated immune response and compromised barrier function play central roles. Thus, vitamin D deficiency in IBD patients not only impacts bone health but may also exacerbate disease activity and severity, highlighting its potential as a modifiable risk factor and therapeutic target [3,4,5].

The clinical relevance of vitamin D is also evident in other malabsorptive gastrointestinal diseases, such as chronic pancreatitis, where vitamin D deficiency, often due to exocrine insufficiency, is a recognized contributor to reduced bone mineral density and increased fracture risk [6]. Low serum levels of vitamin D are frequently observed in individuals with IBD, although it remains unclear whether deficiency is a contributing factor to disease onset and progression or primarily a consequence of chronic intestinal inflammation and malabsorption [7]. Nonetheless, an expanding body of evidence suggests that inadequate vitamin D levels may be associated with a range of adverse outcomes in IBD. Studies have indicated that patients with suboptimal vitamin D status tend to exhibit more severe disease activity, characterized by increased mucosal inflammation and higher clinical disease indices. Furthermore, vitamin D deficiency has been linked to a poorer response to conventional and biologic therapies, potentially undermining the effectiveness of treatment strategies aimed at inducing and maintaining remission. It has also been correlated with a higher risk of IBD-related complications, including strictures, fistulas, and hospitalization. Beyond the direct impact on disease course, low vitamin D levels in IBD patients are associated with diminished overall health outcomes, encompassing reduced quality of life, increased fatigue, and higher rates of comorbidities such as osteoporosis and infections. These findings underscore the potential role of vitamin D not only as a marker of disease severity but also as a modifiable factor that may influence the trajectory of IBD management and patient well-being [8].

This review aims to provide an up-to-date synthesis of the current understanding of vitamin D in the setting of IBD. It discusses the biological underpinnings of its role in intestinal homeostasis, examines the prevalence and clinical implications of deficiency in this population, and considers how addressing vitamin D insufficiency might support disease management and long-term patient well-being.

## 2. Materials and Methods

A comprehensive electronic search of literature published up to April 2025 was conducted using Medline and the Cochrane Library. The search strategy combined Medical Subject Headings (MeSH) and keywords, including: “vitamin D”, “vitamin D deficiency”, “IBD”, “Inflammatory Bowel Disease”, “Crohn’s”, “Crohn’s disease”, “CD”, “ulcerative colitis”, “UC”, “vitamin D receptor”, “VDR”, “inflammation”, “response to therapy”, “therapy”, “biologics”, and “advanced therapy”. Two authors independently screened the titles and abstracts of the retrieved studies to identify those meeting inclusion criteria. Additional relevant articles were identified through manual review of the reference lists of selected papers and review articles. Any discrepancies were resolved by consensus after consulting the sources. This process was conducted as part of a narrative review.

## 3. Vitamin D: Metabolism and Role in Inflammatory Bowel Diseases

### 3.1. Vitamin D: Metabolism, Biological Functions, and Its Potential Role in IBD Pathogenesis

Vitamin D is a fat-soluble secosteroid hormone primarily synthesized in the skin through exposure to ultraviolet B (UVB) radiation, which converts 7-dehydrocholesterol to previtamin D_3_. This molecule then undergoes thermal isomerization to form vitamin D_3_ (cholecalciferol), which, along with dietary sources of vitamin D, is transported to the liver. There, it is hydroxylated to 25-hydroxyvitamin D [25(OH)D], the major circulating form and the most reliable marker of vitamin D status. A subsequent hydroxylation in the kidneys, mediated by the enzyme 1α-hydroxylase (CYP27B1), converts 25(OH)D to its biologically active form, 1,25-dihydroxyvitamin D [1,25(OH)_2_D] [9,10,11].

The biological actions of vitamin D are predominantly exerted through the vitamin D receptor (VDR), a nuclear transcription factor expressed in various tissues, including immune cells and the intestinal epithelium. Upon binding to 1,25(OH)_2_D, VDR heterodimerizes with the retinoid X receptor (RXR), translocates to the nucleus, and modulates the transcription of numerous genes involved in immune regulation, epithelial integrity, and inflammation control [12,13].

In the immune system, vitamin D exerts a multifaceted modulatory role. It inhibits the differentiation and function of pro-inflammatory Th1 and Th17 cells while promoting the development of regulatory T cells (Tregs), thereby contributing to immune tolerance. Moreover, it downregulates the production of key inflammatory cytokines, such as interleukin-17 (IL-17), interferon-γ (IFN-γ), and tumor necrosis factor-α (TNF-α), while enhancing anti-inflammatory mediators like IL-10. These actions are particularly relevant in the context of IBD, where immune dysregulation plays a central role in disease pathogenesis [12,13]. Although CD and UC differ in their underlying immune pathways, with CD being more Th1/Th17-driven and UC involving Th2 and innate responses [14], evidence on the impact of vitamin D on Th17 activity remains limited and somewhat conflicting between the two conditions, warranting further investigation [15].

The pathogenesis of IBD involves a complex interplay of genetic predisposition, immune dysregulation, environmental factors, intestinal dysbiosis, and compromised epithelial barrier function. Emerging evidence suggests that vitamin D exerts modulatory effects on several of these pathological pathways, including immune responses, epithelial integrity, and microbial balance, highlighting its potential as a modifiable factor in the development, progression, and management of IBD [16].

### 3.2. The Role of Vitamin D in Intestinal Epithelial Barrier Integrity in IBD

Vitamin D plays a key role in maintaining the integrity of the intestinal epithelial barrier by upregulating the expression of tight junction proteins, such as claudins, occludins, and zonula occludens (ZO), thereby reducing epithelial permeability and promoting cell survival. Through these mechanisms, vitamin D helps prevent the translocation of microbial products and dietary antigens into the lamina propria, limiting inappropriate immune activation and chronic inflammation. This is particularly relevant in inflammatory bowel disease (IBD), where one of the earliest pathogenic events is the breakdown of the gut barrier. Vitamin D deficiency or impaired signaling through its receptor (VDR) compromises barrier function, contributing to increased intestinal permeability—a hallmark of IBD pathogenesis [11,12].

**Figure 1:**
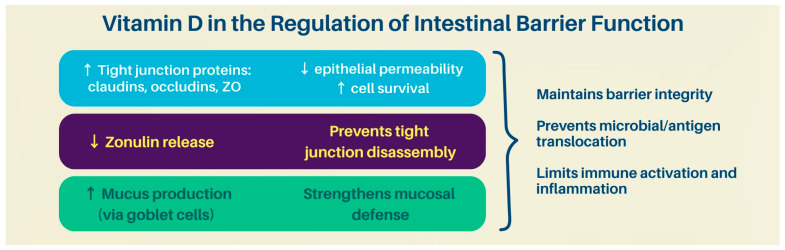
Vitamin D and its mechanisms regulating intestinal barrier function. ZO: zonula occludens.

Vitamin D also regulates the expression of zonulin, a modulator of tight junction disassembly. Elevated zonulin levels, observed in IBD patients, contribute to barrier dysfunction. Vitamin D suppresses zonulin release, reinforcing epithelial cohesion. Furthermore, it promotes mucus production from goblet cells, enhancing the mucosal layer that serves as a frontline defense against microbial invasion. These mechanisms collectively illustrate how vitamin D deficiency can compromise the physical and functional integrity of the intestinal barrier, thereby initiating or amplifying mucosal inflammation [11] (Figure 1).

### 3.3. The Role of Vitamin D on Microbial Dysbiosis in IBD

The disruption of intestinal homeostasis in IBD is further aggravated by microbial dysbiosis. Patients typically exhibit a reduction in beneficial, anti-inflammatory species, such as *Faecalibacterium prausnitzii* and *Lactobacillus*, and an overgrowth of pathobionts like *Enterobacteriaceae* and *Fusobacterium*. Vitamin D plays a crucial role in modulating microbial composition, promoting the growth of commensal bacteria that produce short-chain fatty acids (SCFAs) such as butyrate, known to strengthen the epithelial barrier and suppress pro-inflammatory cytokines [17]. Concurrently, vitamin D reduces the abundance of pathogenic bacteria and enhances the expression of antimicrobial peptides, including cathelicidin and β-defensins, which provide further protection against microbial translocation and dysbiosis-induced inflammation [10,18]. In this context, VDR deficiency may not directly cause dysbiosis but could render the host more vulnerable to microbiota alterations and heightened inflammatory responses following environmental or inflammatory challenges, supporting the concept of a “second hit” mechanism [12,18].

### 3.4. The Role of Vitamin D on the Immune System in IBD

From an immunological perspective, vitamin D is known to shift the immune balance away from pro-inflammatory Th1 and Th17 responses, which are upregulated in IBD, toward regulatory T cells (Tregs) and anti-inflammatory cytokine profiles. It suppresses key mediators of inflammation such as TNF-α, IL-6, IL-17, and IFN-γ, while upregulating IL-10 and TGF-β [12]. These immunoregulatory effects are central to preventing uncontrolled immune activation in response to intestinal antigens. Moreover, experimental studies have shown that VDR-deficient mice develop more severe colitis with increased apoptosis of epithelial cells and exaggerated Th1/Th17 responses, further emphasizing the immunomodulatory role of vitamin D in gut inflammation [19].

The role of the VDR is further supported by studies on single-nucleotide polymorphisms (SNPs), which show that certain SNPs in VDR genes (e.g., ApaI, BglI, and TaqI), associated with reduced efficacy of vitamin D supplementation, have been linked to autoimmune diseases, including IBD [20,21,22].

Taken together, these findings support the hypothesis that vitamin D deficiency may contribute directly to the pathogenesis of IBD by weakening the intestinal barrier, promoting dysbiosis, and amplifying immune dysregulation. While vitamin D deficiency may also result from malabsorption and systemic inflammation associated with IBD, its mechanistic contributions to disease onset and progression position it as both a biomarker of disease severity and a potential modifiable risk factor. Understanding the role of vitamin D in the early molecular events leading to intestinal inflammation offers valuable insight into IBD pathophysiology and may open avenues for preventive or adjunctive therapeutic strategies [23] (Table 1).

## 4. Vitamin D Deficiency in Patients with IBD: Prevalence and Predictors

Vitamin D deficiency is a widespread public health concern, yet universally accepted thresholds for defining deficiency and insufficiency remain lacking. Most clinical guidelines define insufficiency as serum 25(OH)D levels below 20–30 ng/mL (50–75 nmol/L), thresholds primarily based on bone health outcomes [24,25] The World Health Organization adopts stricter definitions, categorizing levels <10 ng/mL as deficient and <20 ng/mL as insufficient [26]. The Endocrine Society, by contrast, recommends higher cut-offs, particularly in the context of chronic diseases and immune function [25]. Importantly, the optimal serum level of 25(OH)D for extra-skeletal effects, especially in relation to inflammation and chronic disease prevention like IBD, remains under active investigation. In this context, it is essential to define not only the lower threshold for vitamin D deficiency but also the upper safety limits of vitamin D status. Several large-scale epidemiological studies have shown that both insufficient and excessive levels of 25(OH)D are associated with an increased risk of all-cause mortality [27]. A retrospective cohort study from Denmark involving over 240,000 subjects identified the lowest mortality risk at serum levels of 50–60 nmol/L, with significantly increased risks at both extremes: 10 nmol/L (HR 2.13) and 140 nmol/L (HR 1.42) [28].

The prevalence of vitamin D deficiency among patients with IBD varies across studies, influenced by factors such as geographic location, study design, population characteristics, and the diagnostic thresholds applied. One of the most comprehensive evaluations to date is a meta-analysis by Sadeghian et al., which included 63 observational studies and reported an overall prevalence of vitamin D deficiency of 57.7% in CD patients. The analysis also showed that individuals with CD had lower serum 25(OH)D levels compared to healthy controls (mean difference −3.99 ng/mL). Notably, latitude was inversely associated with 25(OH)D concentrations, suggesting a potential environmental influence on vitamin D status [29].

A similar trend has been observed in the pediatric population. A recent systematic review and meta-analysis investigating children with IBD reported a pooled prevalence of vitamin D deficiency or insufficiency of 44% (95% CI: 34–54%). Although the difference in mean vitamin D levels between IBD patients and healthy controls (−1.16 ng/mL) did not reach statistical significance, the analysis revealed substantial heterogeneity across studies (I^2^ = 97.3%), suggesting variability in study designs and populations [30].

The higher odds of suffering from vitamin D deficiency in IBD patients compared to healthy controls have been proven in a systematic review and meta-analysis by Del Pinto. The pooled analysis of 14 observational studies (n = 1891) showed that individuals with IBD were 64% more likely to be vitamin D deficient (OR = 1.64; 95% CI: 1.30–2.08; *p* < 0.0001). When analyzed separately, patients with UC had more than twice the odds of deficiency compared to controls (OR = 2.28; 95% CI: 1.18–4.41). Latitude did not significantly influence the association [31].

Multiple factors have been identified as predictors of vitamin D deficiency in patients with IBD. These include high body mass index (BMI > 30 kg/m^2^), non-Caucasian ethnicity [32,33], longer disease duration, increased disease activity, smoking, small bowel involvement, nutritional status, and seasonal variation. Dietary restrictions, common among IBD patients, often reduce vitamin D intake, while limited sunlight exposure during active disease phases or due to a sedentary lifestyle further exacerbates deficiency [34,35,36,37]. Indeed, the higher incidence of IBD in high-latitude regions like North America and Western Europe has been associated not only to lifestyle and environmental factors such as smoking, reduced breastfeeding, increased antibiotic exposure, and improved hygiene but also to limited sunlight exposure due to latitude, which can contribute to vitamin D deficiency [38]. Pharmacologic factors also contribute; for instance, the use of cholestyramine for bile acid diarrhea post-distal ileum resection can impair the absorption of fat-soluble vitamins, including vitamin D [39].

Among vitamin D deficiency predictors, there is also the history of IBD-related surgery, particularly in CD patients [32,33]. However, vitamin D deficiency is also highly prevalent among those UC patients who have undergone total proctocolectomy with ileal pouch-anal anastomosis (IPAA). A large single-center study on patients with UC post-IPAA found that vitamin D deficiency remained common in this population, despite the absence of active pouchitis [40]. These findings are consistent with earlier studies that noted vitamin D deficiency and osteopenia rates of 21.7% and 26% to 55%, respectively [41,42,43], highlighting the persistent risk of malabsorption and nutrient deficiencies after surgery. Following IPAA, vitamin D deficiency is believed to arise from mechanisms such as bacterial overgrowth in the small intestine, enhanced bile salt deconjugation, and compromised chylomicron-mediated absorption [44,45].

The presence of known risk factors and predictors underscores the importance of identifying at-risk patient populations who may benefit from targeted screening and supplementation strategies to address untreated or undertreated vitamin D deficiency (Table 2).

## 5. Clinical Impact of Vitamin D Deficiency in IBD Patients

### 5.1. The Role of Vitamin D in IBD Onset

The role of vitamin D in IBD may begin as early as disease onset, influencing immune regulation and potentially contributing to disease pathogenesis. A systematic review and meta-analysis identified low vitamin D levels as a significant dietary risk factor associated with the onset of IBD. The analysis confirmed that low vitamin D intake, along with low dietary fiber, is linked to an increased risk of developing IBD. Specifically, higher vegetable intake was associated with a 42% reduced risk of IBD overall (RR 0.58, 95% CI 0.53–0.63), with subgroup analyses showing an 87% risk reduction for IBD (RR 0.13, 95% CI 0.04–0.22), 52% for UC (RR 0.48, 95% CI 0.24–0.71), and 18% for CD (RR 0.82, 95% CI 0.76–0.88). Conversely, high intakes of total fats, polyunsaturated fatty acids (PUFAs), and omega-6 fatty acids were associated with increased risk of both CD and UC [46]. In this context, a large prospective cohort study from the Nurses’ Health Study by Ananthakrishnan et al. investigated whether predicted vitamin D status was associated with the risk of developing CD or UC. Among over 72,000 women followed for more than 1.4 million person-years, higher predicted plasma 25(OH)D levels were significantly associated with a reduced risk of incident CD (HR 0.54, 95% CI 0.30–0.99, *p* for trend = 0.02), while the inverse association with UC was not statistically significant (HR 0.65, 95% CI 0.34–1.25). Notably, women with vitamin D levels > 30 ng/mL had a 62% reduced risk of developing CD compared to those with levels <20 ng/mL. These findings support a potential protective role of adequate vitamin D levels in the early phases of IBD, particularly in the pathogenesis of CD [47]. These findings underscore the multifactorial dietary influences on IBD risk, with low vitamin D intake, reduced vegetable and fiber consumption, and high intake of fats all associated with increased disease susceptibility. While vitamin D deficiency may contribute to immune dysregulation and potentially influence disease onset, the broader dietary pattern characteristic of a Western lifestyle likely plays a key role. These insights support the importance of comprehensive nutritional strategies, including both vitamin D optimization and high-fiber, anti-inflammatory diets, in the context of IBD prevention [46].

### 5.2. The Role of Vitamin D on IBD Outcomes

Furthermore, vitamin D deficiency in patients with IBD has been associated with various negative clinical outcomes, including increased disease activity, poor treatment response, higher relapse rates, and a greater risk of both intestinal and extraintestinal complications. A comprehensive systematic review and meta-analysis including 27 observational studies with 8316 IBD patients (3115 with UC and 5201 with CD) demonstrated that low serum 25(OH)D levels are significantly associated with increased odds of clinically active disease (OR 1.53, 95% CI 1.32–1.77, *p* < 0.00001), mucosal inflammation (OR 1.25, 95% CI 1.06–1.47, *p* = 0.008), lower quality-of-life scores (OR 1.30, 95% CI 1.06–1.60, *p* = 0.01), and higher risk of clinical relapse (OR 1.31, 95% CI 1.17–1.47, *p* < 0.00001), with negligible heterogeneity (I^2^ = 0% for all outcomes) [48].

These findings support the frequent occurrence of vitamin D deficiency in IBD and its association with markers of disease activity and adverse clinical outcomes. However, it is important to recognize the bidirectional relationship between vitamin D status and inflammation. While vitamin D deficiency may contribute to immune dysregulation and exacerbate inflammation, chronic inflammatory states may themselves lead to reduced vitamin D levels. Indeed, some studies suggest that vitamin D behaves as a negative acute-phase reactant, with serum concentrations decreasing in response to systemic inflammation [49]. This raises the possibility that low vitamin D levels may, at least in part, reflect underlying disease activity rather than serving solely as a causal factor. Therefore, caution is warranted when interpreting studies linking vitamin D deficiency to disease severity, as reverse causation cannot be excluded. Nonetheless, the consistent association between low vitamin D levels and worse clinical outcomes highlights its potential utility as a biomarker of disease activity and as a modifiable factor in the overall management of IBD. Indeed, several studies have reported correlations between vitamin D status and inflammatory markers in IBD, further supporting its relevance in monitoring disease activity, while also reinforcing the need to distinguish causality from consequence in this complex relationship [49].

An inverse relationship between vitamin D levels and inflammatory markers, including C-reactive protein (CRP), leukocyte counts, and fecal calprotectin (FC), in the IBD population exists [50,51,52]. In a systematic review and meta-analysis by Guzman et al., which included five studies and a total of 253 IBD patients, vitamin D supplementation was associated with a mean reduction of 1.52 points in the Harvey Bradshaw Index (HBI) (95% CI 0.14–2.90; *p* = 0.03; I^2^ = 51%) and a decrease in CRP levels (mean difference 3.70 mg/L; 95% CI 0.10–7.29; *p* = 0.04; I^2^ = 0%) [53].

Complementing these findings, a retrospective longitudinal study by López-Muñoz et al. demonstrated that lower serum 25(OH)D_3_ levels were significantly associated with elevated FC in both CD and UC, as well as increased CRP levels in UC patients. Patients were stratified by vitamin D status into severe deficiency (SD), moderate deficiency (MD), and sufficiency (S) groups. Those with severe deficiency experienced a higher inflammatory burden and poorer clinical outcomes, including more frequent hospitalizations, disease flares, steroid use, and the need for treatment escalation. Moreover, a multivariate model incorporating FC, CRP, and fibrinogen accurately predicted SD status in 80% of cases [50]. A growing body of evidence supports a link between vitamin D status and the risk of clinical relapse in patients with IBD in remission. Several studies have consistently shown that vitamin D deficiency may contribute to disease recurrence, and supplementation could play a preventive role. Gubatan et al. conducted a prospective study on 70 UC patients in clinical remission, revealing that those who experienced relapse within 12 months had significantly lower baseline 25(OH)D levels (mean 29.5 ng/mL) compared to those who maintained remission (mean 50.3 ng/mL; *p* = 0.001). A threshold of ≤35 ng/mL was independently associated with increased relapse risk (OR 1.25; 95% CI 1.01–1.56; *p* = 0.044), with good predictive accuracy (AUC = 0.72), 70% sensitivity, and 74% specificity [54]. These findings are reinforced by a systematic review and meta-analysis by Guzman et al., showing that vitamin D supplementation significantly reduced the risk of clinical relapse compared to controls (OR 0.33; 95% CI 0.18–0.61; *p* = 0.0004; I^2^ = 0%), corresponding to a 67% lower odd of relapse among those supplemented [53]. Similarly, a larger meta-analysis found that vitamin D supplementation was associated with a 36% reduction in relapse risk (RR 0.64; 95% CI 0.46–0.89; I^2^ = 25%). Notably, this effect was more pronounced in patients with CD, with a 53% risk reduction in those in remission (RR 0.47; 95% CI 0.27–0.82; I^2^ = 0%). Data for UC patients were more limited, but trends suggested a comparable benefit [55]. Taken together, these studies provide converging evidence that low vitamin D levels are associated with a higher risk of IBD relapse and that supplementation may be a beneficial strategy, particularly in CD patients in remission.

The impact of vitamin D deficiency extends beyond the gut. Low vitamin D levels have been associated with a higher incidence of extraintestinal complications, such as osteopenia and osteoporosis, particularly in patients on long-term corticosteroids or those with malabsorption [56]. Furthermore, vitamin D plays a role in immune defense, and its deficiency has been associated with an increased susceptibility to infections, including respiratory and opportunistic infections, particularly in immunosuppressed patients [57].

Furthermore, various IBD extraintestinal manifestations have been described as being linked to vitamin D levels. Given its immunomodulatory functions, vitamin D deficiency may play a role in the onset or exacerbation of extraintestinal manifestations frequently seen in IBD, such as psoriasis and arthritis, joint pain, and ocular complications, like uveitis. However, despite these associations, current evidence on the therapeutic benefits of vitamin D supplementation for these extraintestinal symptoms is inconclusive, underlining the need for further investigation [58,59].

Moreover, vitamin D deficiency has been associated with poorer sleep quality in patients with IBD. In one study on CD patients, poor sleep quality, indicated by a Pittsburgh Sleep Quality Index (PSQI) score greater than 5, was present in 61% of patients. Notably, those with vitamin D deficiency had significantly higher PSQI scores (mean 10.5) compared to those without deficiency (mean 6.7; *p* = 0.008), indicating worse sleep quality [60].

Beyond its association with poor sleep, vitamin D deficiency may also contribute to fatigue, one of the most frequent complaints among IBD patients in remission. This link may be partly explained by the presence of vitamin D receptors in skeletal muscle, suggesting that insufficient vitamin D impairs muscle function and could lead to fatigue-related symptoms [61]. In a study analyzing risk factors for fatigue, severe fatigue was reported in 16.7% of cases and was significantly more frequent among those with active IBD, inflammation, and anxiety. Notably, multivariate analysis identified low vitamin D levels and working status as independent predictors of fatigue severity (*p* = 0.052 and *p* = 0.049, respectively), underscoring the potential role of vitamin D deficiency in exacerbating fatigue in IBD [61]. However, a multicenter cross-sectional study involving 405 IBD patients (56% CD, 44% UC) found no significant association between vitamin D deficiency (<50 nmol/L) and either total fatigue or chronic fatigue. While 50% of patients were vitamin D deficient and 29% reported chronic fatigue, fatigue was more strongly linked to higher disease activity, depressive symptoms, and sleep disturbances rather than vitamin D levels [62]. Although these findings suggest a potential role for vitamin D in fatigue, the overall evidence remains inconclusive, and data from human studies are limited and often contradictory. Moreover, despite the high prevalence of vitamin D deficiency in IBD and other chronic conditions, few studies have assessed the impact of supplementation on fatigue as a clinical outcome. These limitations hinder the translation of current evidence into practice and highlight the need for well-designed interventional studies to clarify whether optimizing vitamin D status can meaningfully reduce fatigue in this population [63].

### 5.3. The Role of Vitamin D in Patients with IBD Treated with Advanced Therapy

Recent evidence increasingly supports the role of vitamin D as a key modulator of immune response and a potential predictor of treatment outcomes in patients with IBD receiving biologic therapies. Low vitamin D levels have been associated with reduced response rates to anti-TNF therapy, such as infliximab (IFX) and adalimumab (ADA). This may be due to the immunomodulatory role of vitamin D in promoting a shift from pro-inflammatory Th1/Th17 responses toward a more regulatory immune profile [64,65,66]. In small retrospective studies, vitamin D deficiency has been associated with primary non-response, non-remission, and durability of anti-TNF therapy [64,65,66]. Winter et al. suggest a potential link between vitamin D status and initial response to TNF-α inhibitors since they demonstrated that patients with deficient vitamin D levels prior to initiating TNF-α therapy had a reduced likelihood of achieving remission at three months [64]. Zator et al. concluded that patients with insufficient serum vitamin D levels are more likely to discontinue anti-TNF-α therapy prematurely compared to those with adequate vitamin D status at the initiation of biologic treatment [65]. Furthermore, a retrospective study showed that vitamin D3 supplementation (125 IU/day) in biologic-naïve Chinese CD patients enhanced the effectiveness of IFX, leading to higher clinical remission rates at 54 weeks, particularly in those with vitamin D deficiency. The benefit may be mediated by an upregulation of IL-10 [66]. However, in contrast to smaller studies, findings from the PANTS study (Personalised Anti-TNF Therapy in CD) suggest that baseline vitamin D status may not be a reliable predictor of response to anti-TNF therapy in biologic-naive patients with active luminal CD. In this large prospective cohort, baseline 25(OH)D levels were measured in over 1100 patients treated with IFX or ADA. Although vitamin D deficiency and insufficiency were highly prevalent (17.1% and 47.7%, respectively), pre-treatment vitamin D levels were not associated with either primary non-response at week 14 or non-remission at week 54 for either biologic (*p* values all >0.05). These results challenge previous assumptions by demonstrating that, despite the immunomodulatory role of vitamin D, its baseline concentration does not predict therapeutic outcomes to anti-TNF agents in this well-characterized IBD population [67].

Adequate vitamin D status appears to influence clinical and endoscopic response, particularly in those treated with vedolizumab (VDZ), a monoclonal antibody targeting the gut-homing integrin α4β7. A retrospective study conducted involving 88 IBD patients (44 UC, 44 CD) demonstrated that patients with baseline serum vitamin D levels ≥ 30 ng/mL achieved better therapeutic outcomes. In UC, higher vitamin D levels were associated with significantly lower UCEIS scores after six months of VDZ therapy (1.5 vs. 3.87; *p* = 0.037), reflecting improved mucosal healing. In CD, patients with sufficient vitamin D had notably higher iron saturation (25% vs. 12%; *p* = 0.008), elevated vitamin B12 concentrations (885 vs. 433.5 pg/mL; *p* = 0.003), and increased trough levels of VDZ (27.35 vs. 14.35 μg/mL; *p* = 0.045). Moreover, inflammatory markers such as CRP, HBI, and SES-CD were lower post-treatment in those with higher vitamin D, underscoring its possible role in enhancing both systemic and mucosal control of disease activity. However, it is important to note that improved nutritional parameters, such as higher iron and B12 levels, may themselves reflect a better overall nutritional status, which could act as a confounding factor. Thus, while vitamin D sufficiency may contribute directly to enhanced treatment response, some of the observed effects might also be mediated by or associated with broader indicators of general health [68]. Further supporting these observations, a study employing immunophenotyping and gene expression analyses provided mechanistic insights into the association between vitamin D status and response to VDZ. In a cohort of 48 biologic-naïve IBD patients, higher serum 25(OH)D levels were inversely correlated with the presence of α4β7-expressing immune cells in both peripheral blood (R = −0.400, *p* < 0.01) and intestinal tissues (R = −0.538, *p* = 0.03). This included reduced frequencies of α4β7+ B cells and natural killer cells in the periphery, as well as α4β7+ B cells, NK cells, monocytes, and macrophages in the gut. Transcriptomic analyses of mucosal biopsies from the GEMINI I and GEMINI LTS trials revealed that higher expression of the VDR was inversely associated with the expression of integrin subunits ITGA4 and ITGB7, suggesting that vitamin D may transcriptionally suppress α4β7 integrin expression and thus modulate cellular trafficking to the gut. Clinically, this immunological profile translated into significantly worse outcomes among patients with low baseline vitamin D levels. Specifically, those with 25(OH)D < 25 ng/mL had markedly increased odds of primary non-response to VDZ during induction (OR 26.10, 95% CI 14.30–48.90; *p* < 0.001) and of treatment failure at one year (OR 6.10, 95% CI 3.06–12.17; *p* < 0.001). These findings emphasize that vitamin D status is not merely a marker of nutritional state but may be functionally linked to VDZ efficacy [69].

Beyond vitamin D levels alone, genetic factors related to vitamin D metabolism have also emerged as potential predictors of response to biologics. In a prospective study by De Vita et al., 103 IBD patients (67 with Crohn’s disease and 36 with ulcerative colitis) were genotyped for SNPs in key vitamin D-related genes, including CYP24A1, GC, CYP27B1, and VDR. The study found that ustekinumab (UST) was associated with a higher clinical response at 12 months compared to VDZ (85.7% vs. 67.5%; OR 2.89, 95% CI 1.10–7.60; *p* = 0.03), and early clinical response at three months strongly predicted sustained remission (OR 6.91, 95% CI 2.48–19.30; *p* = 0.0002). Notably, the GC 1296 AC polymorphism was linked to poorer outcomes with VDZ (only 46.7% responders; OR 4.57, 95% CI 1.12–18.73; *p* = 0.04), while the CYP24A1 8620 AG variant was associated with failure to normalize FC levels (*p* = 0.045). Other variants such as CYP24A1 22,776 CT and VDR Cdx2 GG were associated with a greater likelihood of CD over UC (OR 3.40, *p* = 0.009; and OR 3.74, *p* = 0.047, respectively), and certain CYP27B1 genotypes were linked to non-ileal disease (OR 3.13, *p* = 0.054; and OR 7.02, *p* = 0.01) [70].

Taken together, these findings provide strong evidence that vitamin D, through both its serum levels and genetic regulation, plays a key role in modulating response to biologic therapy in IBD. Beyond serving as a marker of nutritional status, vitamin D deficiency may actively contribute to poorer clinical outcomes. Its impact on immune cell phenotypes relevant to agents like VDZ, along with its potential as a biomarker, highlights vitamin D as a promising target for personalized treatment strategies. However, data are still lacking for other advanced therapies and small molecules, underscoring the need for further prospective studies to clarify their therapeutic utility across the full spectrum of IBD treatments (Table 3).

## 6. Strategies for Vitamin D Supplementation in IBD Patients

Addressing vitamin D deficiency in patients with IBD is a critical component of comprehensive disease management, as it may influence both skeletal health and disease-related outcomes. Oral supplementation remains the most common approach, with vitamin D_3_ (cholecalciferol) generally preferred over vitamin D_2_ (ergocalciferol) due to its superior bioavailability and greater efficacy in raising and maintaining serum 25(OH)D levels. Dosage regimens vary widely, reflecting the heterogeneity of IBD populations and individual patient characteristics. Typically, daily low-dose supplementation ranging from 1800 to 10,000 IU is used to restore and sustain adequate levels. Alternatively, high-dose intermittent strategies, such as 50,000 IU administered weekly or biweekly, have been employed to achieve more rapid correction of deficiency, particularly in cases of severe depletion or poor adherence. The choice of dosing strategy is often tailored based on factors such as baseline vitamin D status, the extent and location of disease (notably ileal involvement in CD), BMI, smoking status, and individual absorption capacity. Emerging data suggest that inflammation-related malabsorption, particularly in patients with extensive small bowel involvement or resections, may necessitate higher or prolonged dosing. Despite these considerations, optimal supplementation strategies for IBD patients remain an area of ongoing research, intending to maximize clinical benefits while minimizing risks of hypervitaminosis D [58,71].

Pharmacokinetically, daily and weekly oral dosing, as well as high-dose oral or intramuscular bolus administration spaced months apart, effectively maintain adequate serum 25(OH)D levels. However, intramuscular bolus injections result in a delayed serum increase by about one month compared to oral dosing [72]. Moreover, randomized controlled trials (RCTs) have linked high-dose bolus vitamin D to an increased risk of falls and fractures, and consequently, caution is recommended with high-dose supplementation, favoring daily dosing as the safer approach [73,74,75]. In patients with severe malabsorption, particularly those with extensive small bowel involvement, post-surgical resections including post-IPAA, or active inflammation, oral supplementation may prove inadequate. Given vitamin D’s role in cellular regulation and immune system modulation, as well as the increased risk of low bone mineral density and fractures, particularly in patients with a history of pre-colectomy corticosteroid use, there is a growing rationale for implementing standardized post-IPAA follow-up protocols [31]. In such cases, supplementation with higher doses or parenteral administration of vitamin D may offer a viable alternative, ensuring effective repletion and bypassing the compromised gastrointestinal tract [76].

Nevertheless, high-dose vitamin D supplementation requires caution, as excessive levels have been associated with toxicity, including hypercalcemia, hypercalciuria, renal impairment, nephrolithiasis, and soft tissue or vascular calcification. These risks are particularly relevant with long-term use; therefore, regular monitoring of serum calcium and 25(OH)D levels is essential during extended high-dose regimens [77,78].

In addition to oral supplementation, dietary and behavioral strategies should be considered for maintaining adequate vitamin D levels. Dietary sources from our Mediterranean diet include fatty fish (e.g., salmon, mackerel, and herring) and fortified foods (such as milk, cereals, and margarine), which are particularly relevant for individuals with limited sun exposure. Importantly, the skin is the primary site of cholecalciferol synthesis, a process influenced by several factors, including skin pigmentation, use of clothing or sunscreen, latitude, and season. Encouraging outdoor activities, such as regular walking, not only promotes endogenous vitamin D synthesis but also contributes positively to mental health. Given the limitations of oral absorption in patients with active small bowel inflammation or post-surgery patients, emphasizing dermal synthesis and dietary intake becomes especially important in clinical practice [79,80].

Optimal serum 25(OH)D targets in IBD remain an area of ongoing investigation. Although there is no established gold standard for optimal serum 25(OH)D concentrations in IBD, evidence generally indicates that levels above 75 nmol/L are associated with improved inflammatory markers and clinical outcomes [81], with findings indicating 25(OH)D levels above 50 ng/mL are associated with improved clinical outcomes as opposed to 30 ng/mL in IBD patients [82]. However, considerable variability exists across studies and clinical guidelines regarding optimal serum 25(OH)D thresholds, with extreme heterogeneity depending on the intended clinical outcome and regional guideline differences. As per treatment length, duration of therapy is typically guided by follow-up assessments of serum 25(OH)D concentrations and clinical response [71,83].

**Figure 2:**
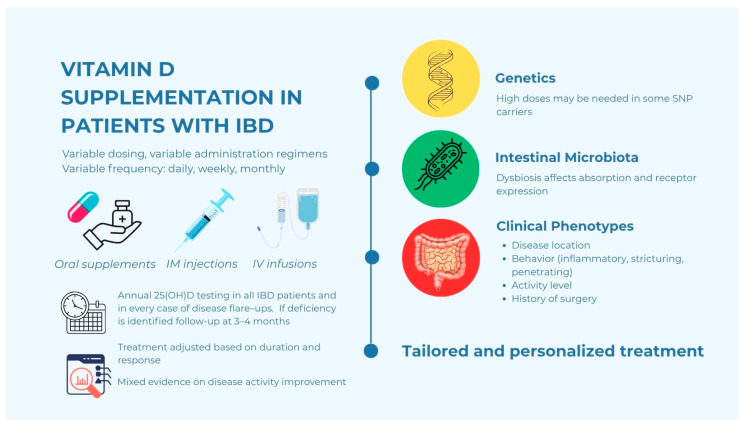
Supplementation of vitamin D in patients with IBD: a tailored approach. The copyright of the figure belongs to the authors.

While most studies and supplementation protocols have focused on adult populations, evidence on pediatric IBD remains more limited. Nonetheless, recent meta-analytic findings suggest that vitamin D supplementation in children and adolescents with IBD, particularly at doses ≥2000 IU and durations >12 weeks, may lead to significant improvements in serum 25(OH)D concentrations, inflammatory markers, and potentially disease activity, with no major safety concerns. However, age-specific protocols and data on long-term effects, including growth outcomes, are still lacking and warrant further investigation [84] (Figure 2).

## 7. Discussion

Vitamin D supplementation has been shown to significantly increase serum 25(OH)D levels in patients with IBD, as demonstrated by a recent meta-analysis of randomized clinical trials. This effect was observed across multiple RCTs, particularly in those with higher doses and longer durations of treatment. Subgroup analyses suggest that lower daily doses administered over extended periods may be more effective in improving vitamin D status. Despite these biochemical improvements, the impact of vitamin D on disease activity and inflammatory markers such as CRP remains inconclusive [85]. Most of the included studies did not find significant reductions in disease activity indices or CRP levels following supplementation. However, a few trials stand out. Notably, an RCT by Yang et al. demonstrated that 24 weeks of supplementation with up to 5000 IU/day led to a meaningful decrease in disease activity scores in CD patients [86]. Likewise, Sharifi et al. observed a reduction in high-sensitivity CRP (hs-CRP) levels in the vitamin D-treated group [87]. A separate comprehensive work found similar anti-inflammatory effects. A meta-analysis of 12 randomized controlled trials (n = 611) and 4 observational studies (n = 359) demonstrated that, in addition to correcting deficiency, vitamin D supplementation was associated with clinical improvement, as reflected by a mean reduction of 1.47 points in the HBI and a decrease in high-sensitivity CRP by 1.58 mg/L, indicating reduced systemic inflammation [88]. Furthermore, in adult IBD patients, supplementation with 40,000 IU of vitamin D every week for 8 weeks has been shown to reduce disease activity indices, FC levels, and serum CRP concentrations, while concurrently increasing albumin levels [89].

These conflicting results may be attributed to variations in baseline inflammatory status, disease stage, and methodological differences among studies—such as inconsistent definitions of vitamin D deficiency, heterogeneity in dosing regimens and supplementation duration, differences in patient populations, and potential confounding factors related to overall poor nutritional status and concurrent micronutrient deficiencies. It is also worth noting that vitamin D’s immunomodulatory properties could contribute to improved clinical outcomes, particularly if supplementation is initiated early in the disease course or targeted to subgroups of patients who may derive the greatest benefit, such as those with severe deficiency or high inflammatory burden. By modulating both innate and adaptive immune responses and promoting epithelial barrier integrity, vitamin D may attenuate mucosal inflammation, reduce disease activity, and enhance the response to standard therapies. However, while current evidence supports the importance of vitamin D in restoring serum levels and suggests potential benefits in disease modulation, more robust and well-designed clinical trials are needed to clarify its therapeutic potential on both inflammatory and clinical parameters in IBD. Future research should aim to identify optimal dosing strategies, timing of intervention, and patient subgroups most likely to benefit from targeted vitamin D supplementation [85].

Emerging approaches emphasize the need for personalized interventions through precision nutrition strategies. These include adjusting supplementation protocols based on genetic polymorphisms affecting vitamin D metabolism, individual microbiota profiles, and specific disease phenotypes. Precision-based approaches aim to optimize therapeutic efficacy while minimizing the risks associated with over- or under-supplementation. Recent advances in nutrigenomics have identified specific SNPs in genes involved in vitamin D metabolism, such as CYP2R1, CYP27B1, GC (vitamin D-binding protein), and VDR. These genetic variants can influence the efficiency of vitamin D hydroxylation, transport, and receptor activation, thereby modulating individual responses to supplementation. For instance, patients carrying polymorphisms associated with reduced enzymatic activity may require higher doses or more frequent monitoring to achieve therapeutic serum levels [9,70]. Supporting this personalized approach, a recent study found that IBD patients in endoscopic remission had significantly higher vitamin D levels than those with active inflammation. Importantly, lower vitamin D levels were linked to homozygosity for the ancestral FokI allele in the vitamin D receptor gene, highlighting a genetic factor contributing to deficiency. However, no differences in other VDR polymorphisms were associated with disease activity, suggesting that vitamin D supplementation can potentially overcome the negative effects of certain genetic variants. This evidence reinforces the importance of integrating genetic profiling into vitamin D management strategies for IBD patients to tailor supplementation effectively. Although vitamin D supplementation can often overcome the effects of certain genetic variants, routine genetic testing may not be necessary in all cases, especially when serum levels can be effectively monitored and corrected. However, genetic profiling may be useful in patients who show a poor response to standard supplementation or present with persistent deficiency despite adequate dosing. In such cases, genetic data can complement serum monitoring to guide more personalized and effective interventions [90].

In addition to host genetics, the composition and function of the intestinal microbiota have emerged as key modulators of vitamin D metabolism and immune regulation. Dysbiosis, a hallmark of IBD, can affect vitamin D absorption and bioavailability, as well as alter the expression of vitamin D receptors in gut epithelial and immune cells. Understanding patient-specific microbial profiles could inform the development of co-interventions, such as prebiotics or probiotics, aimed at restoring microbial balance and enhancing vitamin D efficacy [91]. Moreover, precision nutrition considers the heterogeneity of IBD phenotypes, including disease location, behavior (inflammatory, stricturing, penetrating), and activity level, as these factors may influence both vitamin D requirements and responsiveness. For example, patients with CD involving the ileum, where vitamin D is absorbed, may have greater needs or respond poorly to standard regimens, necessitating individualized dosing or parenteral administration [58,71].

By integrating genomic data, microbiome analysis, and clinical phenotyping, precision-based strategies offer the potential to tailor vitamin D supplementation more effectively than current one-size-fits-all approaches. This paradigm shift recognizes the interindividual variability in vitamin D metabolism, absorption, and immune response, which may be influenced by genetic polymorphisms in vitamin D-related pathways, variations in gut microbial composition, and differences in disease phenotype and severity. By leveraging these data, clinicians may be able to identify patients who are more likely to benefit from specific supplementation strategies or who are at greater risk of deficiency-related complications. This approach not only aims to maximize therapeutic benefits—including improved mucosal healing, reduced disease activity, and enhanced response to therapy—but also seeks to minimize the risks associated with hypervitaminosis D or persistent deficiency. Ultimately, the integration of personalized nutritional interventions represents a promising avenue for optimizing the management of IBD and enhancing patient outcomes.

## 8. Conclusions

Growing evidence supports the notion that vitamin D is not merely a nutritional cofactor but a critical modulator in the pathogenesis, clinical activity, and therapeutic response in IBD. Low serum 25(OH)D levels have been associated with an increased risk of IBD onset, particularly CD, suggesting that vitamin D may play a role in early immune dysregulation and disease development [46,47]. In established IBD, vitamin D deficiency is consistently linked to worse clinical outcomes, including increased disease activity, higher relapse rates, poorer quality of life, and a greater incidence of both intestinal and extraintestinal complications [53]. These associations underscore the role of vitamin D not only as a comorbidity but also as a potential biomarker of disease severity. Supplementation appears to improve both clinical and biochemical markers of disease activity, reduce inflammation, and lower the risk of relapse, particularly in patients with CD in remission [55]. Furthermore, vitamin D status has emerged as a potential predictor of response to advanced therapies. Deficiency has been associated with suboptimal outcomes to anti-TNF agents and VDZ, with mechanistic data suggesting that vitamin D modulates gut-homing immune cell populations and influences integrin expression [64,65,66,68]. Genetic polymorphisms in vitamin D metabolism genes may further impact therapeutic efficacy, offering insights into personalized treatment approaches [70]. Altogether, the evidence highlights the multifaceted impact of vitamin D in IBD, spanning from its potential role in disease onset and progression to its influence on treatment response and overall patient outcomes. Given its immunomodulatory properties and capacity to support epithelial integrity, vitamin D emerges as a critical factor in the complex pathophysiology of IBD. Accordingly, monitoring and optimizing vitamin D status should be considered a key component of comprehensive IBD management, not only to support bone health but also to potentially improve disease control and therapeutic efficacy. Moreover, as biologic and small-molecule therapies continue to expand the therapeutic armamentarium for IBD, further prospective studies are warranted to elucidate and validate the role of vitamin D both as a therapeutic target and as a stratification tool to identify patients who may benefit most from individualized supplementation strategies. In this context, precision nutrition approaches that integrate vitamin D optimization may pave the way for a more tailored and effective management of IBD.

## Figures and Tables

**Table 1:** Vitamin D as a player in intestinal barrier functioning.

Pathogenic AXIS	Vitamin D Role	Consequence of Deficiency/ VDR Impairment
Epithelial Barrier Integrity	↑ Tight junction proteins (claudins, occludins, ZO) via VDR signaling	↓ Tight junction expression → ↑ gut permeability (“leaky gut”) → immune activation
Zonulin Regulation	↓ Zonulin release → maintenance of tight junctions	↑ Zonulin → tight junction disassembly → barrier dysfunction
Mucus Production	↑ Goblet cell activity → ↑ mucosal layer protection	↓ Mucus layer → increased exposure to luminal microbes
Microbial Composition	Promotes growth of SCFA-producing commensals (e.g., *Faecalibacterium*)	Dysbiosis: ↓ beneficial species, ↑ pathobionts (*Enterobacteriaceae*, *Fusobacterium*)
Antimicrobial Peptides	↑ Cathelicidin, β-defensins	↓ Antimicrobial defense → ↑ microbial translocation
Immune Modulation	Shifts from Th1/Th17 → Tregs; ↑ IL-10, TGF-β; ↓ TNF-α, IL-6, IL-17, IFN-γ	↑ Pro-inflammatory cytokines and Th1/Th17 dominance → chronic intestinal inflammation

ZO: Zonula Occludens; VDR: Vitamin D Receptor; SCFA: short-chain fatty acids; ↑ indicates increase, ↓ indicated decrease.

**Table 2:** Risk factors for vitamin D deficiency in IBD patients.

Risk Factor	Notes
High BMI (>30 kg/m^2^)	Associated with lower bioavailability of vitamin D
Non-Caucasian ethnicity	Likely due to reduced cutaneous synthesis from increased melanin
Longer disease duration	Chronic inflammation may impact nutrient absorption
Increased disease activity	Higher inflammatory burden linked to lower vitamin D levels
Smoking	Potential negative effect on nutrient absorption
Small bowel involvement (CD)	Reduces vitamin D absorption
Nutritional status & dietary restrictions	Reduced intake of vitamin D-rich foods
Seasonal variation & reduced sun exposure	Especially during disease flares or in sedentary individuals
Latitude	Higher incidence of IBD in high-latitude countries where sun exposure is reduced
Pharmacologic factors	Impairs absorption of fat-soluble vitamins (e.g., cholestyramine)
IBD-related surgery (e.g., ileum resection, IPAA)	Malabsorption risk persists even in absence of active disease or pouchitis

BMI: Body Mass Index, CD: Crohn’s disease, IBD: Inflammatory Bowel Disease, IPAA: Ileal pouch-anal anastomosis.

**Table 3:** Selected evidence on the role of vitamin D in patients with IBD treated with advanced therapy.

Author	Study Type	Therapy	Population	Key Findings
Winter et al. (2017) [64]	Retrospective	Anti-TNF (IFX, ADA)	173 IBD patients	Low pre-treatment vitamin D levels associated with reduced likelihood of remission at 3 months after starting anti-TNF therapy.
Zator et al. (2014) [65]	Retrospective	Anti-TNF (IFX, ADA)	101 IBD patients	Patients with insufficient vitamin D were more likely to discontinue anti-TNF therapy prematurely.
Xia et al. (2021) [66]	Retrospective cohort	IFX	Biologic-naïve Chinese CD patients	Vitamin D3 supplementation (125 IU/day) improved clinical remission at 54 weeks, especially in vitamin D-deficient patients.
Lin et al. (2023) [67]	Large prospective cohort	IFX, ADA	>1100 patients with luminal CD	Baseline vitamin D status not predictive of primary non-response to anti-TNF at week 14 or non-remission at week 54.
Abraham et al. (2023) [68]	Retrospective	VDZ	88 IBD patients (44 UC, 44 CD)	Vitamin D ≥ 30 ng/mL predicted significant endoscopic improvement in UC patients and lower CRP levels in CD patients.
Gubatan et al. (2021) [69]	Translational + transcriptomic	VDZ	48 VDZ-naïve IBD patients	Low serum 25[OH]D associated with α4β7+ immunophenotypes; vitamin D < 25 ng/mL linked to higher risk of primary non-response and treatment failure at 1 year.
De Vita et al. (2024) [70]	Prospective + genotyping	UST, VDZ	103 IBD patients (67 CD, 36 UC)	SNPs in vitamin D-related genes (e.g., GC, CYP24A1) predicted differential responses to UST and VDZ; specific genotypes linked to poorer outcomes with VDZ.

IFX: Infliximab, ADA: Adalimumab; IBD: Inflammatory Bowel Disease; CD: Crohn’s Disease; VDZ: Vedolizumab; UC: Ulcerative Colitis; UST: Ustekinumab; SNPs: single-nucleotide polymorphisms.

## Abbreviations

The following abbreviations are used in this manuscript:

IBD	Inflammatory Bowel Disease
CD	Crohn’s Disease
UC	Ulcerative Colitis
25(OH)D	25-hydroxyvitamin D
VDR	Vitamin D Receptor
ZO	Zonula Occludens
BMI	Body Mass Index
IPAA	Ileal pouch-anal anastomosis
RCTs	Randomized Controlled Trials
HBI	Harvey-Bradshaw Index
CRP	C-reactive protein
hs-CRP	high-sensitivity CRP
FC	Fecal Calprotectin
IFX	Infliximab
SNPs	Single nucleotide polymorphisms
ADA	Adalimumab
VDZ	Vedolizumab
UST	Ustekinumab
